# Exploring the Social Environment with the Eyes: A Review of the Impact of Facial Stimuli on Saccadic Trajectories

**DOI:** 10.3390/ijerph192416615

**Published:** 2022-12-10

**Authors:** Mario Dalmaso

**Affiliations:** Department of Developmental and Social Psychology, University of Padova, 35121 Padova, Italy; mario.dalmaso@unipd.it

**Keywords:** saccadic trajectory, visual attention, faces, eye movements, social cognition

## Abstract

Eye movement parameters can be highly informative regarding how people explore the social environment around them. This theoretical review examines how human faces and their features (e.g., eye-gaze direction, emotional expressions) can modulate saccadic trajectories. In the first part, studies in which facial stimuli were presented in a central location, such as during a face-to-face social interaction, are illustrated. The second part focuses on studies in which facial stimuli were placed in the periphery. Together, these works confirm the presence of an intriguing link between eye movements and facial processing, and invite consideration of saccadic trajectories as a useful (and still underused) opportunity to track ongoing mechanisms that support the social vision. Some directions for future research are also discussed.

## 1. Introduction

The environment in which human beings live and interact is intrinsically social. In everyday life, our visual system is consistently exposed to numerous inputs from our conspecifics, and several studies have reported that human attention is profoundly shaped by social stimuli, especially faces and eye gaze [1,2]. Even if social stimuli can covertly orient our attention (i.e., without eye movements; see, e.g., [3,4,5]), during social interaction, we usually perform several eye movements to keep track of the signals provided by others. For these reasons, the study of eye movements in social contexts is of great interest as it can provide relevant insights concerning how human beings interact with each other. Moreover, there exists a rich universe of eye movements that can offer a more direct index of attentional allocation over space as compared with other behavioural measures (e.g., manual responses; [6]).

Different eye movements and eye-related measures have been used to investigate the impact of facial stimuli on visual attention, such as pupil size (e.g., [7]) or fixational eye movements (i.e., microsaccades; e.g., [8]). However, most studies on this topic have employed saccadic eye movements, with particular emphasis on saccadic latency and accuracy (e.g., [9,10,11,12,13,14,15,16,17,18]). Much less is known about a separate, yet still relevant, statistic of saccades: their trajectories. In fact, it is known that when an individual performs a saccade, its path is rarely a straight line between the starting point and the endpoint, rather it presents a curved trajectory that can be modulated by ongoing attentional mechanisms [19,20]. Although evidence of saccadic curvatures can be found in the pioneering work of Yarbus (1987) [21] (see also [22]), the first specific study of saccadic curvatures was conducted by Sheliga et al., (1994) [23]. In their study, a central cue prompted participants to covertly orient their attention towards a peripheral object, and then a saccade had to be performed towards a target. Saccades tended to deviate away from the location to which attention was allocated. More recently, it has been observed that saccades deviated away even from task-irrelevant distractors presented in the periphery [24], and that this curvature tended to be greater for particularly salient distractors, such as in the case of distractors that were perceptually similar to the target [25,26]. When combined, these studies suggest that saccades tend to deviate away both from an attended location and from an irrelevant stimulus within the visual field. According to some authors, saccadic trajectories are caused by the activity within the saccade map contained in the superior colliculus (SC; [19,27,28]). More precisely, inhibitory mechanisms would occur within this map to mitigate the potential impact of distracting stimuli on planned eye movement, thus causing a deviation in the saccadic trajectory [19]. In line with this notion, a particularly salient distracting stimulus would require greater inhibition; consequently, this would lead to a greater deviation away from the spatial location associated with the distractor. Deviations towards the location associated with the distractor can also be reported, but this would occur by adopting, for instance, some specific paradigms, such as the double-step task or during a visual search [20]. Deviations towards a certain location can be reported even when saccades with relatively short latency (i.e., roughly less than 200 ms) are taken into account [29,30], suggesting that the inhibitory mechanisms underlying saccadic curvatures require time to fully emerge.

### Taking Advantage of Saccadic Trajectories

The evidence discussed in the previous paragraph suggests that saccadic trajectories can be used (1) to track attention allocation over space, such as when a central signal is provided [23], and (2) to assess the impact of peripheral distractors during target selection [24]. Although the mechanisms underlying attention allocation and target selection can be explored through more standard saccadic parameters, such as latency and accuracy [9,31], saccadic trajectories can offer some useful advantages. First, saccade trajectories occur without awareness, and their parameters (curvature direction and amplitude) cannot be controlled volitionally. This allows penetrating deeply into the mechanisms supporting face processing which can be influenced by social beliefs (e.g., [32]). Second, while saccadic latency and accuracy are discrete events and, therefore, can provide only limited information on the mechanisms involved in visual processing, saccadic trajectories evolve within a space–time continuum, allowing for an assessment of visual processing at several stages [33,34,35]. In other words, saccadic trajectories might be considered a more informative index compared with both latency and accuracy. Third, and more related to social contexts, some previous studies employing central gaze stimuli found modulatory effects of social variables more clearly reflected in accuracy (i.e., directional errors) than in latency analyses [9,12,15,16]. However, the percentage of these directional errors is typically low (e.g., ≤5–10% of trials) and therefore saccadic trajectories allow one to look at all of the data rather than at a subset. Fourth, since saccadic trajectories strongly depend on SC activity [27,28], they could be a particularly suitable oculomotor index when exploring mechanisms that recruit subcortical pathways involving SC, such as eye-gaze or facial expression processing [36,37]. Consistent with this notion, a recent study reported smaller latencies for saccades directed toward a face with a direct gaze than an averted gaze [14], confirming that direct-gaze faces (i.e., faces making eye contact) can capture attention (see [36,38,39]; see also [40,41,42]). However, the pattern of results reported in [14] only emerged when ‘express saccades’ (i.e., saccades with a latency of approximately 80–120 ms; see, e.g., [43]) were considered—which are known to depend on SC [44]. When express and regular saccades were analysed together, no face-based effects emerged [14]. This could tentatively explain why most studies exploring the impact of facial stimuli on saccadic trajectory (see the next paragraph) did not report face-based effects analysing saccadic latency.

Other oculomotor measures can provide a continuous measure to track attentional allocation over space, such as the size of the pupil [45]. However, the pupil response to cognitive modulations can require seconds to fully emerge [7,45], while the effects of social stimuli on attentional mechanisms are generally fast rising and fast decaying [15,41]. For example, it is known that a face with an averted gaze can elicit attentional shifts towards the same location (i.e., gaze cueing of attention [3,4,5,32,46,47]), but these shifts can be detected within a relatively narrow time window after averted-gaze onset (i.e., roughly 100–1000 ms; [46]). This suggests that saccadic trajectories would be preferable to pupil size when investigating the temporal dynamics underlying social orienting.

In the next paragraph, the impact of facial stimuli on saccadic trajectories will be illustrated, while the concluding paragraph will provide advice on how saccadic trajectories might help in solving some empirical issues concerning social orienting mechanisms.

## 2. Saccadic Curvatures Modulated by Facial Stimuli

Despite the pervasive effects of facial stimuli on both visual attention and eye movements [1,9], few studies have explored the potential impact of these highly relevant social stimuli on saccadic curvatures. These studies can be practically divided into two categories: those in which the facial stimulus was presented in the centre of the screen, and those in which the facial stimulus was presented in a peripheral location. Studies within the first category have mainly explored attention orienting in response to eye-gaze cues, whereas studies within the second category investigated the effects of peripheral faces acting as distractors.

### 2.1. Centrally Placed Facial Stimuli

**Figure 1:**
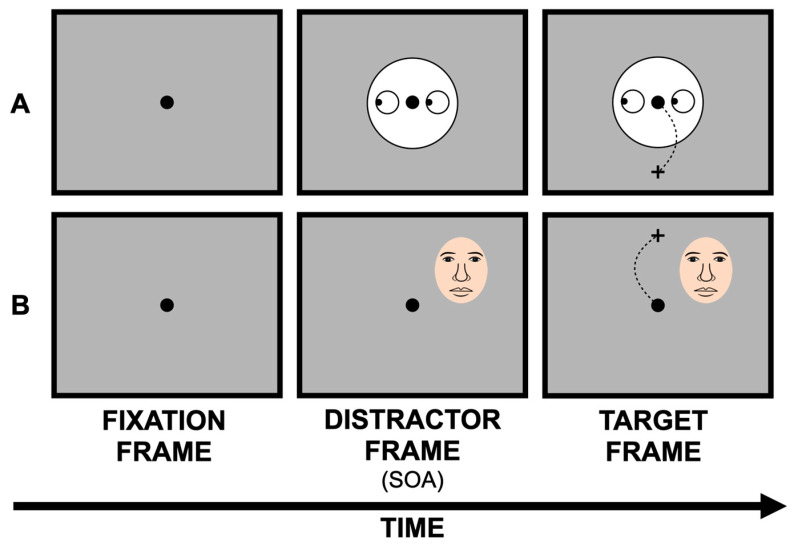
Examples of the two main paradigms used to explore the impact of facial stimuli on saccadic curvatures. A central fixation spot is typically followed by a distracting face and a symbolic target stimulus. In (**A**), a central distracting face with its eye gaze averted leftwards is presented, while (**B**) shows a peripheral distracting face with a direct gaze. The dotted lines represent potential saccadic trajectories. Stimuli are not drawn to scale. SOA = stimulus onset asynchrony.

The use of central faces has two main advantages: first, it mimics what typically happens during a social interaction between two individuals looking at each other; second, it allows researchers to easily manipulate eye-gaze direction to elicit a gaze cueing effect [3,4,5], and the first study exploring the link between faces and saccadic curvatures was based on this notion (i.e., Nummenmaa and Hietanen, 2006; [48]). In more detail, in [48] each trial started with a centrally placed schematic face with its gaze averted leftwards or rightwards (i.e., see also Figure 1, Panel (A)). After a stimulus onset asynchrony (SOA) of either 100 or 0 ms (i.e., simultaneously), participants were instructed to perform a vertical saccade towards a target that could appear either on the upper or lower part of the screen. The results demonstrated that at both SOAs, saccades deviated away from the location cued by the gaze. Importantly, when the pupils of the eyes were removed, and a peripheral black square appeared to the left or right side of the screen, deviations away from such peripheral stimulus were still observed. However, this emerged more clearly at the 0-ms SOA, thus suggesting that facial stimuli should be processed differently from a symbolic peripheral distractor. As for saccadic latencies, no relevant results emerged. The different impact of social and symbolic stimuli on saccadic curvatures was then further explored by Hermens and Walker (2010) [49] in four experiments. Similarly to the previous study [48], participants were presented with a centrally placed face pointing either leftwards or rightwards. This was compared with two other non-social stimuli, namely an arrow oriented leftwards or rightwards and a peripheral distractor appearing leftwards or rightwards. After different SOAs (i.e., 10 ms, 100 ms, or 300 ms), a target appeared either at the top or at the bottom of the screen, requiring participants to perform a vertical saccade towards it. Saccadic trajectories were calculated as average peak deviations (see [49]). The main results can be summarised as follows: at the shorter SOAs (i.e., 10 ms and 100 ms), the saccades deviated away from the peripheral distractor and towards the spatial location indicated by the arrow, even if the latter effect was minimal. On the other hand, the face stimulus led to non-significant modulations. At the longer SOA (i.e., 300 ms), saccades tended to deviate away from the location indicated by all three stimuli, but the curvatures were smaller for both arrow and facial stimuli compared with the peripheral distractor. As in [48], analyses of vertical saccades latencies did not reveal any relevant pattern of results. To conclude, [49] indicates that both arrows and faces can lead to similar modulatory effects on saccadic curvatures, according to the idea that these two stimuli would elicit comparable behavioural effects on visual orienting (see, e.g., [10,17,50]).

Finally, a work by West et al., (2011) [51] manipulated facial expressions, which are known to deeply shape different oculomotor measures [52]. In [51], participants were presented with a central face with a fearful or neutral expression. Then, a target appeared at either the top or bottom of the screen, alongside a peripheral symbolic distractor. Unlike both [48] and [49], in [51], eye-gaze direction was not manipulated, as the central face was always presented with a direct gaze. However, because emotions activate subcortical pathways, including SC [37], the authors reasoned that this potential activation could affect saccadic trajectories. Contrary to this hypothesis, similar saccadic curvatures emerged in the presence of both fearful and neutral faces. However, evidence for shorter saccadic reaction times emerged when fearful, but not neutral faces, were removed 200 ms before target onset. The authors concluded that emotional faces would be able to modulate the temporal (i.e., latency)—but not the spatial (i.e., trajectory)—dynamics of saccades. However, some of the studies discussed in the next section reported an affective modulation of saccadic curvature when emotional faces were presented peripherally rather than centrally.

### 2.2. Peripherally Placed Facial Stimuli

When we perform an eye movement within complex social environments, it is highly likely that many different individuals fall into our peripheral vision rather than centrally. Hence, investigating saccadic curvatures in the presence of peripheral faces is relevant and complementary to studies that employed centrally placed stimuli.

Evidence for a modulation of peripheral facial stimuli on saccadic curvatures has been reported by Laidlaw et al., (2015) [53]. In this work, participants received the instruction to look at a fixation spot and to perform a vertical saccade towards a target placed either in the upper or in the lower part of the monitor; a peripheral distracting face could also appear along with the target (see also Figure 1, panel (B)). The results illustrated that while no differences emerged between faces presented upright or upside down, saccades curved away from upright faces more strongly than from scrambled faces (i.e., abstract images obtained by a random permutation of the pixels constituting the original face images; scrambled faces had therefore the same low-level properties of the original facial stimuli but lacked social relevance). Interestingly, the difference between scrambled and non-scrambled faces was much more evident for saccades with a greater latency, likely suggesting that this face-based effect would require time to emerge. No effects of distractor type emerged in saccadic latency analyses. Qian et al., (2015) [54] employed a paradigm similar to that used in [53]. However, in [54], participants were presented with distracting peripheral faces presented upright or upside down and depicting either the participant’s own face or the faces of unknown individuals. Saccadic curvatures were not modulated either by face orientation (i.e., upright vs. upside down; see also [53]) or by facial identity. However, when the initial direction of the saccades was analysed—instead of their curvatures—a stronger deviation away from the upright faces was reported compared with the upside-down faces, and this held true both when saccades were elicited endogenously (i.e., through a schematic arrow indicating either up or down) and exogenously (i.e., through the target appearing either up or down). Again, facial identity did not play any modulatory role, even if evidence of a stronger face inversion effect emerged for the unknown faces, but only when downward saccades with greater latency were considered. As in [53], the results of saccadic latencies were not modulated by distractor type. Overall, these results are inconsistent with several studies that reported greater covert attentional orientation in response to the participant’s own face or faces that were familiar to them compared with unknown faces (e.g., [55]). More recently, Dalmaso et al., (2017) [56] explored the potential impact of eye contact on saccadic curvatures, revealing that saccades curved away from a direct-gaze face more strongly compared with a face presented with closed eyes or a scrambled face. Furthermore, this difference was more pronounced at longer latencies and when facial stimuli were presented 100 ms before the target onset. The effects on saccadic latencies were negligible.

The role of affective contexts in shaping saccadic curvatures has also been explored. In a first study, Schmidt et al., (2012) [57] instructed participants to perform endogenous vertical saccades according to the direction of a central arrow cue. Simultaneously with the arrow onset, distracting peripheral stimuli also appeared, consisting of neutral objects (e.g., a house) and an upright vs. an upside-down face with a neutral, happy, or angry expression. Overall, saccades curved away more strongly from angry faces compared with the other conditions (i.e., neutral and happy faces or objects), but only when angry faces were presented upright rather than upside down. In particular, saccadic curvatures did not differ between neutral and happy faces compared with objects, a result that contrasts with the previously discussed studies in which facial stimuli elicited greater curvatures than non-facial stimuli (i.e., scrambled faces; see [53,56]). However, it is important to note that in [57], although no significant results emerged from saccadic latencies, their distribution was not taken into account. Therefore, one could speculate that in [57] a face-based effect could have emerged if saccades with higher latency were analysed separately from saccades with shorter latency (see also [53,54,56]). Evidence for greater saccadic curvatures away from angry faces—compared with happy faces—has also been reported by Petrova and Wentura (2012) [58], but this emerged only for upright faces and not for upside-down faces, and for downward rather than for upward saccades. Again, saccadic latency analyses led to irrelevant results. Together, both [57] and [58] reinforce the idea that our oculomotor system is particularly sensitive to negative stimuli (see also [59,60,61]).

## 3. Discussion and Future Directions

The studies discussed in this theoretical review provide supporting evidence for the notion that facial stimuli can shape saccadic trajectories, while both latency and accuracy analyses provided less informative results. On the one hand, a centrally placed face with an averted gaze would cause a saccadic deviation towards the opposite location as that indicated by the gaze direction [48,49], but this deviation would be quantitatively similar to that caused by a central symbolic spatial cue such as an arrow (see [49]). This would confirm that eye gaze and arrow stimuli can elicit similar orienting, at least at the behavioural level (see also, for example, [10,17,50]). As a further step, it would be important to manipulate the social characteristics of the central face (e.g., age, physical dominance, group membership, etc.) to assess whether such characteristics can shape saccadic trajectories (see also [32]). To date, only one study has employed real faces—with direct gazes—displaying fearful or neutral expressions, but this affective modulation led to a null effect on saccadic trajectories [51].

On the other hand, all the studies that employed peripherally placed distracting faces used stimuli depicting real individuals [53,54,56,57,58], obtaining a variety of results. These results can be roughly summarised into the following: saccades tend to deviate more strongly away from a face compared with a non-social scrambled face [53,56], from a face that establishes eye contact with the observer rather than a face looking elsewhere [56], from an angry face compared with neutral and happy faces [57,58], and from an unknown face rather than from the participant’s own face [54]. Face orientation (i.e., upright vs. upside down) led to mixed results, since both a null effect [53] and a greater initial direction away from upright faces than upside-down faces have been reported (see [54]; see also [57,58] for evidence with negative expression). However, in the latter case, differences in the way saccadic trajectories were calculated and the inclusion of the saccade direction (i.e., upward vs. downward, [58]) as an independent variable could explain these divergent results. When used in conjunction, these studies confirm the notion that our attentional mechanisms are sensitive to facial signals coming from the environment [1].

Two other intriguing facts about saccadic trajectories emerged from studies that presented distracting faces in the periphery. First, greater face-based effects have been reported for saccades with longer latencies as compared with saccades with shorter latencies [53,54,56]. This is in line with the notion that the inhibitory mechanisms underlying saccadic deviations need time to arise [29,30] and, in turn, to interact with social variables. Second, greater face-based effects also emerged for downward saccades as compared with upward saccades [54,58]. A tentative explanation for this latter evidence can be found in [58]: because the lower and upper visual hemifields would be associated with the near peri-personal space and the far extra-personal space, respectively (e.g., [62]), downward eye movements would be much more sensitive to any stimulus carrying action (e.g., a graspable tool) or social (e.g., a face) relevance, in order to prepare our body for potential interaction with that stimulus.

Due to the limited number of studies exploring the impact of facial stimuli on saccadic trajectories, several issues can be addressed in the near future. For example, other relevant social stimuli known to shape oculomotor parameters could be used, such as human bodies [63] or self-related stimuli [64,65], and saccadic trajectories could also be recorded in real social interactions by employing wearable eye trackers (see also [66,67]) to increase the ecological validity of the results. Furthermore, the use of saccadic trajectories could also provide relevant insights into some controversial issues regarding social orienting. In this regard, gaze-mediated covert orientation has been found to not be present in individuals with autism spectrum disorder (ASD; see [68]; see also [1]), while eye-tracking data indicated a comparable oculomotor behaviour between healthy individuals and ASD individuals when saccadic latency and accuracy were analysed ([69]; see also [70]). Nevertheless, one might speculate that overt orienting differences between these two groups could emerge by looking at saccadic trajectories. This would find support, although indirect, even within some of the literature that describes a link between autism and SC (see [71,72] for reviews). In fact, SC would be highly involved not only in the design of saccadic trajectories [19] but also in the development of neural networks underlying social behaviour ([71,72], see also [73]). In addition, it might be worth using saccadic trajectories to investigate the potential role of facial expression on the gaze-cueing effect. In fact, available studies on this topic provided mixed evidence (see [32] for a review). For example, a pioneering study found no effect on the gaze-cueing effect [74] (see also [75]), whereas other studies found greater attentional orienting for negative expressions, but only under specific circumstances, such as visual search tasks [76] (see also [77]). Because SC would also be involved in emotion processing [37], saccadic trajectories could represent a sensitive measure to uncover a link between affective contexts and this form of social orientating. Finally, the inhibitory mechanisms elicited by facial stimuli could be further explored by taking advantage of the inhibitory nature of saccadic trajectories. For example, the tendency to inhibit attentional orienting towards previously explored locations (i.e., inhibition of return, IOR; [78]) has received little interest in the literature on social attention using eye-gaze stimuli, and the results are far from conclusive. Indeed, so far gaze-mediated IOR has emerged only under specific circumstances involving a high percentage of non-target trials [79] and particularly long SOAs between cue and target onsets [79,80]. Hence, saccadic trajectories could hopefully reveal novel insights into both the nature and the temporal dynamics of this inhibitory mechanism.

## 4. Conclusions

Saccadic curvatures can be considered a reliable and direct index of attention allocation in the presence of facial stimuli. Future studies employing this oculomotor parameter are needed as they can reveal novel insights into the functioning of social attention.
